# Signed Connectedness Among Cryptocurrencies, NFTs, and Foreign Exchange Markets

**DOI:** 10.3390/e28040439

**Published:** 2026-04-13

**Authors:** Shuang Yang, Xu Zhang, Wenting Xu

**Affiliations:** School of Management Science and Engineering, Nanjing University of Information Science & Technology, Nanjing 210044, China

**Keywords:** cryptocurrencies, NFTs, foreign exchange markets, signed network topology

## Abstract

Existing approaches mainly characterize connectedness in four dimensions: node size, direction, spillover magnitude between nodes, and time variation. However, the sign of spillovers has not been explicitly incorporated into the analysis. To address this limitation, this paper introduces sign as a fifth dimension and constructs a five-dimensional signed network-topology framework based on BVAR historical decomposition. The framework is used to examine the evolution of spillover signs and to explore the multidimensional spillover effects among the cryptocurrency, NFT, and foreign exchange markets. The results show that the signed spillovers across these three markets vary over time during the sample period. On average, DXY exerts negative spillover effects on the cryptocurrency market, while ETH exerts more pronounced negative spillover effects on the NFT market. Furthermore, during the COVID-19 pandemic and the cryptocurrency crash period, BTC shifted from a net receiver of risk spillovers to a net transmitter. In terms of sign, spillover magnitudes vary relatively little across periods, whereas sign reversals occur more frequently.

## 1. Introduction

The origin of complex network theory can be traced back to Euler’s “Konigsberg bridge problem” in the 18th century, which originated from graph theory. The mathematical foundations of complex network research were established with the proposal of stochastic networks in the late 1950s. Later, researchers proposed the small-world and scale-free network models, which greatly advanced complex network research and promoted its application in various fields. Complex network theory is a useful tool for understanding the structure and evolution of complex systems. According to Diebold and Yilmaz [1], the economic and financial system is also a large complex system, and its pluralism, nonlinearity, and systematization have led to the extensive application of complex network methods by scholars. Financial network analysis applies complex network theory to financial systems by constructing networks from financial correlations. It has become an important research direction in financial analysis because it helps reveal the structure and dynamic changes of financial markets and explains and predicts the possible outcomes of interactions among different financial entities.

In recent years, the volatility spillover framework developed by Diebold and Yilmaz [1,2,3], which is based on forecast-error variance decompositions in VAR models, has been widely used to study shock transmission across financial markets. Its main advantage is that it provides a unified way to characterize the direction, magnitude, and time variation of connectedness in a network setting. Existing DY-based network analyses therefore mainly focus on four dimensions, namely node size, direction, spillover magnitude between nodes, and time variation. However, because forecast-error variance decomposition is variance-based, it cannot distinguish whether the underlying transmission is associated with positive or negative signed contributions. As a result, conventional DY networks can describe how strongly markets are connected, but they are less informative about whether spillovers amplify or offset one another. To address this limitation, this paper introduces sign as a fifth dimension of the network topology and uses historical decomposition results to identify the signed spillovers among the cryptocurrency, NFT, and foreign exchange markets.

This paper focuses on the interconnectedness among the cryptocurrency, NFT, and foreign exchange markets. Cryptocurrencies are digital assets built on cryptographic and decentralized blockchain technology, and their prices are largely determined by market demand and supply. Because they are not issued by a central bank and are traded in highly speculative environments, cryptocurrencies are often characterized by substantial volatility. Like cryptocurrencies, NFTs are also digital tokens. But unlike fungible or interchangeable cryptocurrencies, NFTs are non-fungible and unique and can be used to prove the authenticity and uniqueness of digital assets. Through blockchain technology, NFTs can record ownership and transaction history, which enhances the traceability and verifiability of digital assets. In the early days of the NFT market, price behavior was closely linked to the crypto market, but as it matured, price fluctuations became increasingly independent. The decentralization and relative anonymity of cryptocurrencies may reduce their direct exposure to some forms of foreign exchange regulation and exchange-rate risk, thereby creating potential linkages with the foreign exchange market. These three markets have attracted increasing attention in recent economic and financial research.

This paper contributes to the existing literature in several ways. First, the dynamic network-topology framework adopted in this paper incorporates sign into the conventional four-dimensional connectedness structure, so that positive and negative spillover effects can be distinguished within the same network. Second, the paper provides a more comprehensive perspective by jointly examining the cryptocurrency market, the NFT market, and the foreign exchange market. With BTC and ETH representing the cryptocurrency market, ENJ and MANA representing the NFT market, and DXY and INVEUR representing the foreign exchange market, the analysis captures spillover relations among these three markets within a unified framework. Third, the sample period from 2020 to 2023 covers several episodes of pronounced market turbulence, allowing examination of how spillover roles evolve during periods of sharp market fluctuations. Different from studies that focus on asymmetric, nonlinear, or regime-dependent spillovers, this paper emphasizes the sign of cross-market transmission within a unified connectedness framework. This perspective enables identification of whether spillovers are amplifying or offsetting over time, thereby providing information not directly available from approaches that primarily distinguish spillovers across regimes or market states.

The main findings can be summarized as follows. First, representative indicators are selected according to the strength of intra-market volatility spillovers. Based on the historical decomposition results, BTC, as the representative of the cryptocurrency market, has a more pronounced effect on ENJ, the representative of the NFT market, whereas the other markets are less affected by DXY, the representative of the foreign exchange market. Second, the network topology constructed from the average historical decomposition results indicates that the foreign exchange market exerts a positive spillover effect on the NFT market and a negative spillover effect on the cryptocurrency market, except for some weak positive links. As the NFT market gradually developed and became less dependent on the cryptocurrency market, it began to exert a negative spillover effect on that market. In average spillover analyses over the full sample period, the cryptocurrency market, represented by Bitcoin, typically acts as a recipient rather than a transmitter of volatility spillovers. In contrast, the opposite pattern is observed during the pandemic and the cryptocurrency slump. These findings help clarify the risk-return characteristics of different asset classes and shed light on the evolution of volatility spillovers across markets.

The rest of this article is structured as follows. The Section 2 reviews the relevant literature. The Section 3 gives an overview of econometric methods. The Section 4 describes the data. The Section 5 provides empirical results. The Section 6 presents the conclusion.

## 2. Literature Review

A considerable body of the literature has examined the cryptocurrency market. This section reviews relevant studies on the cryptocurrency market itself, the links between the cryptocurrency and NFT markets, the links between the cryptocurrency and foreign exchange markets, and the network-topology method.

### 2.1. Node Size as the First Dimension of Network Topology

In a topological network, nodes represent elements, and node size indicates each element’s influence. Based on the concept of variance decomposition in vector autoregression, Diebold and Yilmaz [1] developed and studied accurate measures of return spillovers and volatility spillovers. The proposed network-topology method is often used to study risk spillover effects in commodity markets [4,5]. With the development of relevant research, scholars have also used the network framework to explore net spillovers between the financial and the commodity markets. For example, Yoon et al. [6] applied the DY method and found that the US stock market is the most important source of spillovers among major Asia-Pacific stock markets. In research on stock-market networks, some scholars found that only the American stock market could trigger systemic risk on a global scale through the size of the network’s nodes and edges, as indicated by empirical results, and others demonstrated a significant volatility spillover effect between the world and Europe. The economic determinants of shock spillover intensity appear to be stronger in Europe than in the world [7,8]. As the US dollar (USD) is the main pricing currency of international crude oil trading, it is widely accepted that there is a strong connection between the oil price and the US dollar exchange rate. Treating the US dollar exchange rate and the oil price as two nodes, many scholars have analyzed the correlation between their fluctuations. On the one hand, some studies have proved that the volatility spillover effect is not significant through cointegration, VAR models, ARCH models, and Granger risk causality, indicating that the price fluctuation takes a relatively independent path, and the immediate fluctuation of the US dollar exchange rate will not cause significant changes in the oil market. On the other hand, some studies have revealed that the fluctuations of the US dollar exchange rate and crude oil price are segmented in the long run, and the degree of dependence is generally weak, but it is time-varying, and the correlation is high before and during the financial crisis and after the recent European debt crisis [9,10].

### 2.2. Direction as the Second Dimension of Network Topology

In network-topology methods, direction refers to the relationship between explanatory variables. The network-topology method proposed by Diebold and Yilmaz [1] has been widely used to study the risk spillover effects in the commodity market, but it has some limitations. Therefore, Diebold and Yilmaz [2] further decomposed the forecast-error variance using a generalized VAR model. This approach effectively addresses the problem of variable dependence and explicitly incorporates directional volatility spillovers. Subsequently, further studies were conducted based on the directional spillover measure proposed by Diebold and Yilmaz [2]. Although scholars pursue different research subjects, they have continually improved their understanding of the directional links between them. For example, one study used a newly introduced set of implied volatility indices to examine the directional connectedness between oil and equities in eleven major stock exchanges around the globe from 2008 to 2015, and found that most of the correlation was dominated by transmission from the oil market to the stock market rather than the other way around [11]. In exploring the net spillover network between financial and commodity markets, Yoon et al. applied the network spillover approach to perform static and dynamic analyses, quantifying net spillover effects and assessing the net directional connectivity of each market [6]. There are also further studies on the bidirectional volatility relationship between variables. Some studies examine different cryptocurrencies using the daily returns of Bitcoin, Ether, and Litecoin and find a bidirectional volatility relationship among the three; others use corn and ethanol as the main body and find a significant bidirectional volatility spillover effect between the two. Moreover, directional volatility spillovers can provide a clearer account of the relationship between variables [12,13].

### 2.3. Spillover Magnitude Between Nodes as the Third Dimension of Network Topology

The spillover effect between nodes can be understood as the magnitude of their influence, expressed by the thickness of the lines in the topological network. With further advances in research, the spillover effect among multiple variables has also attracted the attention of scholars. Among them, Zhao used MGARCH to find the two-way volatility spillover effect between two markets in China [14]. In exploring the static and dynamic spillover effects between commodities and commodity currencies, Yip et al. analyzed the spillover correlation between commodities and commodity currencies from the perspectives of returns and volatility [15]. By analyzing the nature of inter-market mean and volatility transmission mechanism in two G-7 markets, Yang and Doong found that stock price volatility affects exchange rates and generates an asymmetric volatility spillover effect [16]. Many scholars also analyze volatility spillover effects across various multivariate subjects. Some scholars examine spillover effects on the prices of selected agricultural products when foreign exchange and oil prices fall, and find a strong internal link and a weak correlation between oil shocks and agricultural products. It is confirmed that the Indian equity sector index has time-varying differential dependence on oil price fluctuations [17,18]. Some scholars have also studied volatility spillover effects between multiple gold markets and four precious metal markets [19,20], as well as between 21 stock markets across four regions, including Europe and Asia [21].

### 2.4. Time Variation as the Fourth Dimension of Network Topology

Affected by natural disasters, financial crises, war, and other factors, the relationship between variables in an economic system often shows time-varying characteristics. Initially, due to some defects in the general VAR model, Diebold and Yilmaz proposed a rolling-window VAR model that incorporates the idea of time-varying spillover connectivity, and variance decomposition was used to define weighted and oriented networks [3]. Related studies also consider the dynamic properties of spillovers and systemic linkages in different markets and settings [22,23,24]. With the progress of research, more and more scholars began to pay attention to the network-topology extension method proposed by Diebold and Yilmaz. For example, a new method was proposed within the MF-VAR framework to address the availability limitation of time series due to different frequency sampling [25]. In addition, the TVP-VAR model was developed to address the limitations of the rolling window method for estimating the spillover index. Nyakurukwa and Seetharam argue that the TVP-VAR method is advantageous because it eliminates outliers that can skew results and reduces bias caused by selecting arbitrary rolling Windows [26]. The TVP-VAR method has been used to estimate the interconnectivity of crude oil and agricultural assets in a time-varying manner [17]. Cogley and Sargent argue that, under the general VAR framework, the coefficients and the variance-covariance matrix of the error term remain unchanged, which is inconsistent with the law of the real economy [27]. Therefore, TVP-VAR is used to analyze changes in the inflation rate and the natural unemployment rate across different periods. The parameters in the TVP-VAR model are time-varying, but the covariance structure is ignored. Based on this method, Primiceri further improved the TVP-VAR model by assuming that the covariance of the error term also changed with time [28]. Subsequent studies showed that the traditional linear VAR model assumes that the underlying economic structure does not change. Hence, the model parameters remain unchanged even as the external environment changes. However, during the research process, scholars found serious problems with linear analysis. They believed that nonlinear methods could capture the characteristics of nonlinear and structural changes in macroeconomic time series, and were subsequently frequently applied to the measurement of policy effects and economic fluctuations. Some scholars used the asymmetric network-topology method based on the STVAR model to explore different network structures among state variables, and then used the STVAR-based network to estimate the systemic risk spillover network of the commodity market under the bullish and bearish regimes [29,30].

### 2.5. The Correlation of Cryptocurrency Market, NFT Market, and Foreign Exchange Market

A body of the literature studies the interconnectedness of cryptocurrency markets [24,31], for example, using BEKK-MGARCH to analyze the volatility dynamics and interlinkages among major cryptocurrencies [11,32], using a nonlinear Markov switching specification to confirm that cyberattacks have a significant negative impact on the likelihood of cryptocurrencies remaining in a low volatility state [33], and applying the ST-GARCH model to analyze asymmetric volatility among cryptocurrencies [34]. More studies have also examined four major cryptocurrencies, using two different long-memory methods (R/S analysis and fractal integration) to study their random properties, thereby providing a more comprehensive analysis [35]. With the rising value of cryptocurrencies in the market and their increasing popularity worldwide, most studies focus on the market behavior and investment outlook of cryptocurrency markets [36,37,38].

Numerous studies have examined how the cryptocurrency market interacts with other markets. It is generally believed that spillovers from larger markets into smaller markets are a common phenomenon [39]. In studying spillover effects between cryptocurrencies and NFTs, it is argued that the larger cryptocurrency market will affect the growth and development of the smaller NFT market, but there is no reverse effect [40]. It is also indicated that volatility transmission between NFTs and cryptocurrencies is low. However, the wavelet coherence suggests that there is some common movement between the two sets of markets [41]. From a short- and long-term perspective, liquidity spillover between the cryptocurrency and foreign exchange markets is variable over time [42]. Regarding the relationship between cryptocurrencies and foreign exchange rates, some scholars have observed a significantly lower degree of dependence between the two, as estimated by the copula model [43]. In addition to these variance- and copula-based approaches, some studies adopt information-theoretic tools rooted in complexity science to characterize cross-market linkages. For example, Jang et al. use transfer entropy to uncover nonlinear and asymmetric information flows between Bitcoin and major investment assets such as equities, gold, and the US dollar [44], while García-Medina and Hernández C construct a multivariate transfer-entropy network of cryptocurrencies and show how episodes of market turbulence reshape the topology and intensity of cross-asset spillovers [45].

To sum up, most existing studies focus either on the internal dynamics of the cryptocurrency market or on the links between cryptocurrencies and one external market at a time. Relatively few studies evaluate the dynamic volatility spillovers among the cryptocurrency, NFT, and foreign exchange markets within a unified signed framework. More importantly, existing asymmetric, nonlinear, or regime-dependent approaches mainly distinguish spillovers across market states, whereas the present paper focuses on the sign of spillovers itself and thus identifies whether cross-market transmission is reinforcing or offsetting over time. These considerations motivate the present study.

## 3. Methodology

To empirically analyze the dynamic relationships among the cryptocurrency market, the NFT market, and the foreign exchange market, we construct a signed network-topology method. Compared with the existing literature, this paper uses historical decomposition based on a BVAR to construct connectivity across markets.

### 3.1. Signed Network-Topology Model

The application of network theory in economics was initially proposed by Allen and Gale [46]. Later, network theory was widely used to study the correlation among economic variables. Diebold and Yilmaz [1] provide a new perspective on economic networks by combining the VAR model with network-topology theory. The VAR-based network-topology approach proposed by Diebold and Yilmaz includes four dimensions: node size, direction, spillover effect between nodes, and time variation [1,2,3]. In this paper, we introduce a fifth dimension: sign, which is based on the results of historical decomposition. The historical decomposition technique can be explained by reference to the moving average representation of vector autoregression:

(1) $$Y_{t}=A\left(L\right)\eta_{t}=\sum_{s=0}^{\infty }A_{s}\eta_{t-s}$$

For a particular period t+j, $Y_{t+j}$ may be decomposed into the following:

(2) $$Y_{t+j}=\sum_{s=0}^{j-1}A_{s}\eta_{t+j-s}+\sum_{s=j}^{\infty }A_{s}\eta_{t+j-s}$$

The first term on the right-hand side shows the difference between the actual series and this “base projection” due to innovations in the variables after the period t. The second term on the right-hand side represents the dynamic forecast or “base projection” of $Y_{t+j}$, given the information available at the time t. Thus, the gap between each data series and its base projection can be assessed in terms of the contributions of the innovations to each series in the analysis. The base projection and the contributions of the innovations constitute the data.

Now assume $d_{y_{1}\to y_{2}}$ ($d_{y_{1}\to y_{2}}\geq 0$ or $d_{y_{1}\to y_{2}}<0$) is the decomposition value of $y_{1}$ to $y_{2}$, where the historical decomposition results may be either positive or negative, and $y_{1T}$ is the total value of the factorization for $y_{1}$ over the other variables, then the explanation ratio of $y_{1}$ to $y_{2}$ can be expressed as follows:

(3) $$D_{y_{1}\to y_{2}}=\frac{d_{y_{1}\to y_{2}}}{y_{1T}}$$

However, due to the dimensional problem between the numerator and denominator in Equation (3), $D_{y_{1}\to y_{2}}$ may be distorted when the denominator value is extremely small. Therefore, we use Equation (4) to compress the proportional relation. This transformation only compresses the scale of the explanation ratios while preserving the sign and relative ordering of the original decomposition results.

(4) $$d_{12}^{Tz}=\arctan(D_{y_{1}\to y_{2}})=\arctan\left(\frac{d_{y_{1}\to y_{2}}}{y_{1T}}\right)$$

Table 1 presents the spillover matrix among variables. For $d_{12}^{Tz}$ in this table, its sign and value can be positive or negative, which is the most significant difference compared with the existing VAR-based spillover matrix.

The *FROM* column in Table 1 represents the spillover of other variables to $Y_{j}$:

(5) $$S_{FROM,j\leftarrow }^{T,z}=\sum_{i=1}^{N}d_{ij}^{T,z},\;i\neq j$$

The *TO* row in Table 1 represents the spillover of $Y_{i}$ to other variables:

(6) $$S_{TO,\leftarrow i}^{T,z}=\sum_{j=1}^{N}d_{ij}^{T,z},\;i\neq j$$

Element $S^{T,z}$ in the lower right corner of Table 1 measures the total spillover effect among variables. $S^{T,z}$ is calculated as follows:

(7) $$S^{T,z}=\frac{\sum_{ij=1}^{N}d_{ij}^{T,z}}{N},\;i\neq j$$

### 3.2. Historical Decomposition Based on BVAR

To implement historical decomposition, this paper constructs a VAR model. VAR is a non-structural equation system model that is not based on economic theory but instead uses multiple equations simultaneously. In each equation of the model, endogenous variables regress on lagged terms of all endogenous variables. Then, the dynamic relationships among all endogenous variables are estimated, which is often used to predict interrelated time-series systems and to analyze the dynamic impact of random disturbances on the system. The general form of the VAR model is as follows:

(8) $$Y_{t}=c+F_{1}Y_{t-1}+F_{2}Y_{t-2}+\ldots +F_{s}Y_{t-s}+u_{t},\;t=s+1,\ldots ,n$$

where $Y_{t}$ is a k×1 dimensional column vector, c is the intercept vector of k×1, $F_{1}$,…, $F_{s}$ is a matrix of k×k coefficients, and $u_{t}$ is the independent k×1 dimensional normal random exogenous disturbance term. $u_{t}$ satisfies: $i.i.d.N_{k}(0,\Sigma ),\;t=1,2,\ldots ,n$. Σ is a positive definite matrix of k×k. For simplicity of expression, *n* equations can be reduced to a more compact matrix form:

(9) $$Y=ZB+U,\;U:N_{n\times k}(0,\Sigma ⊗I_{n})$$

where the following applies:

(10) $$Y={\left(\begin{array}{l} {Y\prime }_{1}\\ {Y\prime }_{2}\\ M\\ {Y\prime }_{n} \end{array}\right)}_{n\times k}\;\;\;Z={\left(\begin{array}{l} {Z\prime }_{1}\\ {Z\prime }_{2}\\ \ldots \\ {Z\prime }_{n} \end{array}\right)}_{n\times (kp+1)}\;\;\;Z_{t}={\left(\begin{array}{l} 1\\ Y_{t-1}\\ \ldots \\ Y_{t-p} \end{array}\right)}_{(kp+1)\times 1}\;\;\;U={\left(\begin{array}{l} {U\prime }_{1}\\ {U\prime }_{2}\\ \ldots \\ {U\prime }_{n} \end{array}\right)}_{n\times k}$$

VAR is highly parameterized, with the number of parameters increasing proportionally to the number of variables multiplied by the square of the number of lags considered. Excessive parameters increase the variance of forecast errors. Thus, it is recommended to use fewer parameters and more sample data in VAR models to improve model performance. In contrast, Bayesian VAR utilizes both the prior distribution of the model parameters and the sample information to calculate the posterior distribution of the estimated parameters. This approach can provide reliable estimation performance even with small sample sizes, mitigate parameter overestimation, and enhance forecast accuracy. Litterman developed a BVAR model based on the Bayesian estimation method [47]. In the BVAR model, the prior distribution is shown as in Equation (11):

(11) $$p\left(B,\Sigma \right)\infty {\left|\Sigma \right|}^{-0.5(k+1)}$$

Then the posterior distribution of the parameters of the unrestricted VAR model is expressed as follows:

(12) $$p(B\left|Y\right.,Z):Mt_{m\times k}(\hat{B},Z^{\prime }Z,n-m),p(\Sigma \left|Y,Z\right.):IM(S,n-k)$$

where m=kp+1, $\hat{B}={\left(Z^{\prime }Z\right)}^{-1}Z^{\prime }Y,S=Y^{\prime }\left[I_{k}-Z{\left(Z^{\prime }Z\right)}^{-1}Z^{\prime }\right]Y$, $Mt_{m\times k}$ refers to the matrix with *t* distribution, $IM_{k}$ represents the inverse Wishart distribution.

In the empirical implementation, the BVAR is estimated for six endogenous variables, namely BTC, ETH, ENJ, MANA, DXY, and INVEUR. The baseline specification uses one lag, while a two-lag specification is considered later in the robustness analysis. We adopt a Minnesota-style prior mean structure in which the own first lag is centered at 1, and the remaining lag coefficients are centered at 0. In the baseline specification, the prior shrinkage parameter is set to 0.35, the lag-decay parameter to 1.0, and the intercept prior scale to 100. Posterior draws are obtained from the matrix-normal inverse-Wishart posterior distribution, and only stable draws are retained. The historical decomposition is then computed draw by draw, and the resulting signed contributions are used to construct the signed spillover measures analyzed in the following sections.

## 4. Data

### 4.1. Data Source

To examine the signed dynamic spillover network among the cryptocurrency market, the NFT market, and the foreign exchange market, this paper selects two representative indicators for each market. The cryptocurrency market is represented by BTC and ETH. BTC is the most widely recognized cryptocurrency, and its market dominance reflects the overall movement of the cryptocurrency market. ETH ranks second in market capitalization and serves as another important benchmark in cryptocurrency trading. The NFT market is represented by ENJ and MANA. The foreign exchange market is represented by the US dollar index (DXY) and the euro index (INVEUR). DXY reflects the external value of the US dollar against a basket of major currencies, while INVEUR captures the value of the euro in the foreign exchange market.

The sample consists of daily data from 1 January 2020 to 1 May 2023. BTC, ETH, ENJ, and MANA prices are obtained from CoinGecko, while DXY and INVEUR are collected from British Financial Intelligence.

Because the three markets do not share an identical trading calendar, the baseline series are aligned on common observed dates before estimation to ensure that the spillover measures are constructed from synchronized observations. This treatment is particularly relevant in the joint analysis of cryptocurrency, NFT, and foreign exchange markets, whose trading calendars differ across weekdays, weekends, and holidays. To ensure that the main findings do not depend on a particular daily matching rule, the analysis is later complemented by a weekly common-calendar specification in the robustness tests.

### 4.2. Stationarity Test

To avoid the pseudo-regression problem that may be caused by non-stationary series in the model, the time series data are tested for stationarity before empirical analysis. However, because NFT prices were very small in the early stage of market development, each index was multiplied by 1000 to facilitate the subsequent calculations. In this paper, the logarithms of BTC, ETH, ENJ, MANA, DXY, and INVEUR are scaled up in the same proportion and then filtered. The ADF and PP tests are performed on the cycle components after data processing, and the results are shown in Table 2.

Table 2 shows that the original series are non-stationary under the ADF and PP tests, whereas the processed cycle components are stationary at the 5% significance level. We do not differ the data here because differencing would reduce the smoothness of the series and make the results more abrupt. Based on the stationarity test, we construct a BVAR model using stationary series. According to the AIC and SC criteria, the optimal model lag is 1. Furthermore, we observe that all the eigenvalues of the AR eigenvalue test fall within the unit circle, indicating that the estimated BVAR model is stable. Therefore, it is reasonable to use the estimation of the BVAR model to implement the historical decomposition.

## 5. Empirical Results and Policy Implications

### 5.1. Historical Decomposition Results

**Figure 1:**
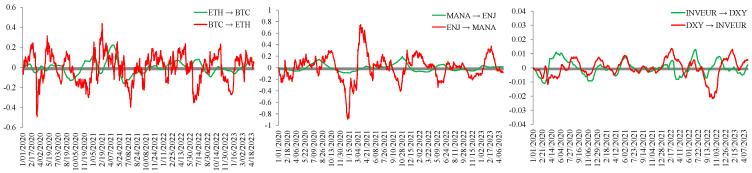
Comparison of spillover intensity across markets using internal indicators.

In the cryptocurrency market, we first analyze the relationship between BTC and ETH. According to Katsiampa et al. [12], there is a two-way transmission of shocks between BTC and ETH, and this paper focuses on the variation in spillover signs over the sample period. The results show that BTC has a stronger effect on ETH. As a store of value and trading tool, BTC’s value is closely related to its position as the world’s largest digital currency. Its market share and circulation make it the dominant player in the cryptocurrency market. Hence, choosing BTC can more clearly reflect the trend of the cryptocurrency market. In the NFT sector, according to Naeem et al. [48], ENJ has the highest volatility, followed by MANA. ENJ has a significant impact on MANA. It is also one of the earlier representative tokens in the NFT sector and reached the highest market value among all NFT tokens in March 2021. In the foreign exchange market, the stronger influence of DXY may reflect the dominant role of the US dollar in the international monetary system. The dollar index measures the strength of the US dollar against a selected basket of currencies. As shown in Figure 1, the dollar index also has a stronger impact on the euro index. To facilitate the subsequent comparative analysis of interactions across markets, BTC, ENJ, and DXY are selected as representative indicators of the cryptocurrency market, the NFT market, and the foreign exchange market, respectively, for further analysis of cross-market linkages.

The cryptocurrency market, the NFT market, and the foreign exchange market are linked through several channels. First, investor crossover enables diversification across markets such as digital currencies, NFTs, and foreign exchange. Some investors may allocate part of their funds to mainstream cryptocurrencies such as Bitcoin, while others may invest in the NFT market or even the foreign exchange market. Second, risk contagion implies that market volatility and risk may be transmitted across different markets [49,50,51]. For example, global economic instability or major changes in the foreign exchange market could have a ripple effect on the digital currency and NFT markets, resulting in price volatility. Third, there is overlap in market participants. Although each market has its own specialized players, many common participants influence all three markets. The interaction between these markets may be influenced by investors’ investment behavior and sentiment [52,53].

**Figure 2:**
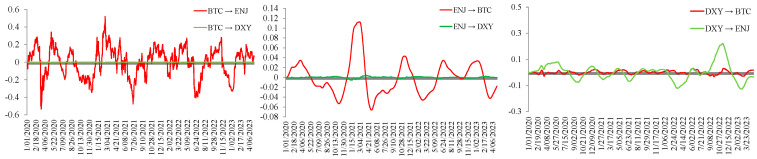
Spillover effects from one market to other markets.

The historical decomposition results of the BVAR model constructed for the representative indicators of the cryptocurrency market, the NFT market, and the foreign exchange market are further analyzed below. In Figure 2, positive values indicate positive signed spillovers, whereas negative values indicate negative signed spillovers. Cryptocurrencies are fungible, standardized, and highly divisible tokens, whereas NFTs are indivisible, non-fungible, and unique. Although the two markets differ in characteristics, the historical decomposition results show that BTC exerts a strong influence on ENJ in the early stage. As the NFT market gradually became more independent, this influence persisted, but its intensity weakened. Compared with the volatility spillover from ENJ to the BTC market, the volatility spillover from BTC to ENJ is more pronounced. When analyzing the spillover effects of BTC and ENJ on DXY, it is clear that the foreign exchange market is less affected by fluctuations in other markets, which is consistent with the study of Drożdż [54]. This pattern may be related to differences in market liquidity, trading activity, and regulatory conditions across markets. First, compared with the foreign exchange market, the cryptocurrency market has lower liquidity and trading volume. Therefore, price fluctuations in the cryptocurrency market may have a limited impact on the foreign exchange market. Second, regulatory and legal provisions in different countries impose different degrees of trading restrictions on cryptocurrencies and NFTs, which may also limit cross-market spillovers. However, the DXY spillover to ENJ is relatively strong. As an important turning point, 2020 brought substantial shocks and uncertainty to the global foreign exchange market owing to the impact of the COVID-19 pandemic. The foreign exchange market from 2020 to 2023 was in a transition period, and the resulting financial market volatility also imposed substantial shocks on investors. Under such conditions, instability in the foreign exchange market may be associated with stronger spillovers to the NFT market.

### 5.2. Topology Network Results with Signed Dimensions

**Figure 3:**
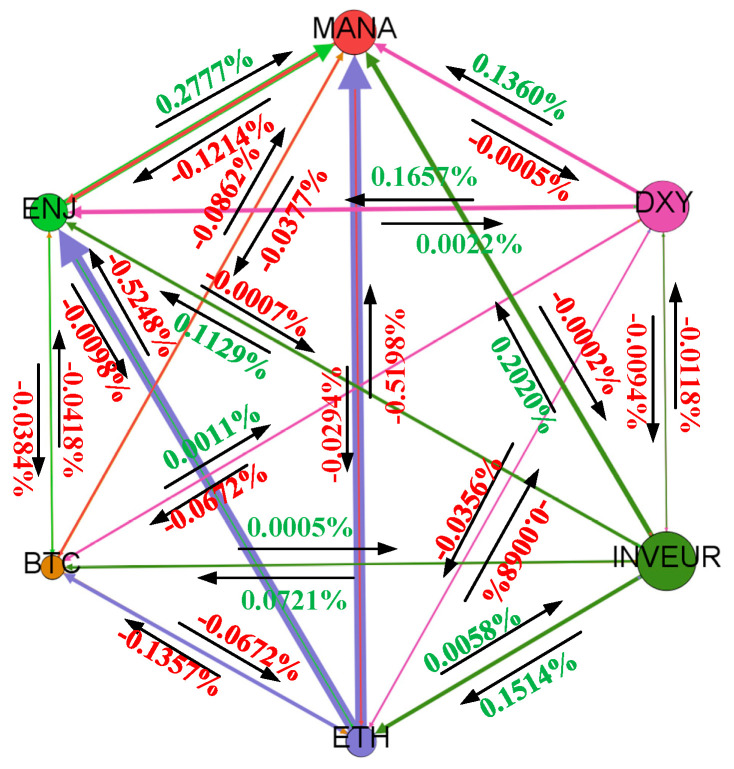
Average signed spillover network for the full sample period.

Figure 3 presents the average signed spillover network for the full sample period. The spillover effects among BTC, ETH, ENJ, MANA, DXY, and INVEUR are analyzed below. In the network figures, arrows indicate the direction of spillover transmission, and the percentages next to the links report the corresponding signed spillover values. Positive values are shown in green, whereas negative values are shown in red. First, we focus on the spillover effects of the foreign exchange market on the variables in other markets. INVEUR has a positive spillover effect on the NFT market. Since the NFT market emerged later, its volatility appears to be more easily influenced by other variables. Although the early volatility of the NFT market depended on the volatility of the cryptocurrency market, the volatility spillover effect of INVEUR on the two markets is different. INVEUR has a weak positive spillover effect on BTC in the cryptocurrency market and a relatively significant negative spillover effect on ETH. DXY also has a clear positive spillover effect on the NFT market and a clear negative spillover effect on the cryptocurrency market. This suggests that spillover effects from the foreign exchange market differ between the cryptocurrency and NFT markets. However, as the NFT market gradually developed and became more independent, the volatility spillovers between the two markets were not identical.

Second, we note that the volatility spillover effect of BTC on other markets is weak, which is consistent with Xu et al. [55], who found that Bitcoin is the largest recipient of systemic risk, while spillovers from other markets to the cryptocurrency market are more pronounced. However, the negative spillover effect of ETH on the NFT market is more significant. ETH shows a relatively stronger degree of market independence, although the presence of other currencies may also affect spillover effects from fluctuations in other markets. Finally, we observe that the NFT market has a clear negative spillover effect on the cryptocurrency market. Unlike the initial development of the NFT market, with the gradual strengthening of its market characteristics and the gradual improvement of market independence, the volatility dependence between the NFT market and the cryptocurrency market has gradually weakened, and the volatility spillover of the NFT market itself can also have an impact on the cryptocurrency market to a certain extent. ENJ has a slight positive spillover effect on DXY and a slight negative spillover effect on INVEUR, but the spillover effect values for both are very small. In contrast, MANA has a weak negative spillover effect on the foreign exchange market.

### 5.3. Spillover Effects of Market Volatility During the Outbreak Period

The outbreak was not only a global health emergency, but also heightened global economic uncertainty [56]. In this context, financial markets reacted sharply and adversely [57], accompanied by a substantial increase in global financial market risks [58,59,60]. Heightened fear and uncertainty may have affected investor sentiment and portfolio allocation decisions [61,62], and these changes may have been reflected in stock prices across markets and spillover effects [63]. We focused on the outbreak period from the perspective of Li et al. [63] and defined the period from 13 February 2020 to 19 August 2022 as the outbreak period to analyze volatility spillover effects within this time frame.

**Figure 4:**
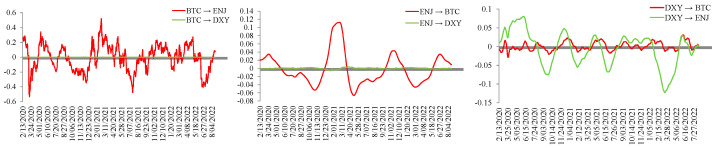
Spillover effects from each market to the other markets during the outbreak period.

The sign changes in the spillover effect of the cryptocurrency market during the outbreak period are relatively significant. During this period, the spillover effect of the cryptocurrency market on the NFT market declines as the dependence of the NFT market on the cryptocurrency market gradually decreases, and the spillover effect value is significantly larger than that of the NFT market on the cryptocurrency market. During the outbreak period, the cryptocurrency and NFT markets acted more as transmitters than as receivers relative to the foreign exchange market, and their spillover effects on the foreign exchange market showed limited fluctuation. By contrast, the sign of the volatility spillover effects of the foreign exchange market on the cryptocurrency and NFT markets changed over time, and the impact of the foreign exchange market on the NFT market was stronger. Figure 4 shows the spillover effects from each market to the other markets during the outbreak period.

Focusing on the fifth dimension, namely sign, we find that the magnitude of spillover effects changes little across different periods, whereas the signs of spillover effects change more frequently. In 2020, ETH shifted from a negative to a positive spillover effect on BTC, and MANA’s spillover effect on BTC also showed sign changes over time. These results suggest that, although the magnitude of changes in the digital currency market during the epidemic was not very pronounced, the sign of the spillover effect reversed. There is a two-way sign reversal effect between DXY and BTC. These results indicate substantial variation in signed transmission during the outbreak period. The negative spillover sign from ETH to ENJ changed, and the bidirectional spillover effect between ETH and MANA also experienced sign reversals.

**Figure 5:**
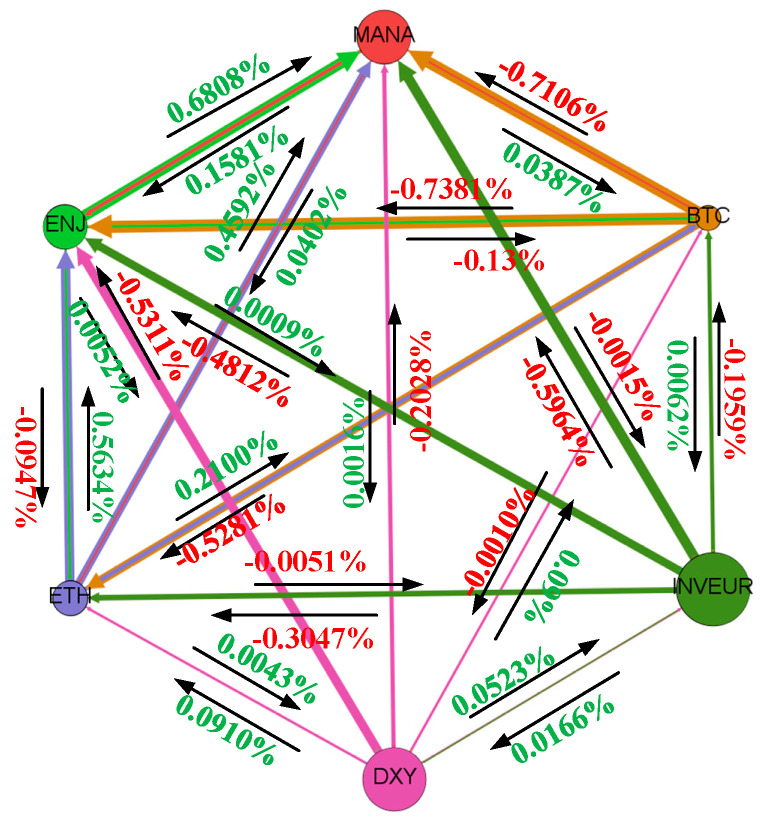
Average signed spillover network during the outbreak period.

In 2021, the NFT received considerable attention. During the total sample period, MANA had a negative effect on ENJ, but during the outbreak period, the estimated effect between MANA and ENJ became positive. The results also showed that the effect of DXY on ENJ during the outbreak period was different from that in the full sample period. The positive spillover observed in the whole sample period turned negative during the outbreak period, while the spillover effect between INVEUR and ENJ showed a two-way sign reversal. The spillover effect between MANA and DXY showed a two-way sign reversal; the spillover effect of INVEUR on MANA turned from positive to negative; and the signed spillover relationship between the NFT market and the foreign exchange market also changed during the outbreak period relative to the full sample. There is also a two-way signed spillover between DXY and INVEUR within the foreign exchange market. Figure 5 shows the average signed spillover network during the outbreak period.

### 5.4. Spillover Effects of Market Volatility During the Cryptocurrency Slump

Based on market developments, we define the period from 12 November 2021 to 20 January 2022 as the cryptocurrency slump period. On November 10 of that same year, the market reached its highest point, after which prices declined rapidly amid tighter regulatory supervision in countries such as China and the United States. Following the rejection of a proposal for a Bitcoin spot ETF by financial regulators on November 12, the price of Bitcoin began to drop.

**Figure 6:**
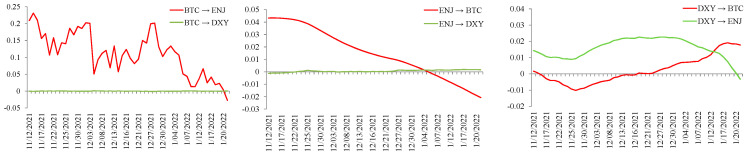
Spillover effects from each market to the other markets during the cryptocurrency slump.

In the context of the collapse of the cryptocurrency market, the NFT market was also affected, with prices declining in the same month. This is also consistent with the positive spillover effect of BTC on ENJ shown in the figure. In the later period, the spillover of the two markets showed a temporary sign reversal, and the negative spillover value was weak. However, the impact of the cryptocurrency and NFT markets on the foreign exchange market remains limited. During the study period, the impact of the foreign exchange market on the cryptocurrency market basically maintained a positive spillover effect with slight fluctuations, which first weakened and then declined gradually after a subsequent increase. The spillover sign remained positive for most of the period and turned negative on 19 January 2022. The time variation in the sign of the spillover effect of the foreign exchange market on the NFT market is more significant. On 25 November 2021, the NFT market reached one of its price peaks and then began to decline. At the same time, the foreign exchange market exerted a relatively strong negative spillover effect on the NFT market. This effect later gradually turned positive, with the sign reversing on 16 December 2021, and then continuing to increase. Figure 6 shows the spillover effects from each market to the other markets during the cryptocurrency slump.

**Figure 7:**
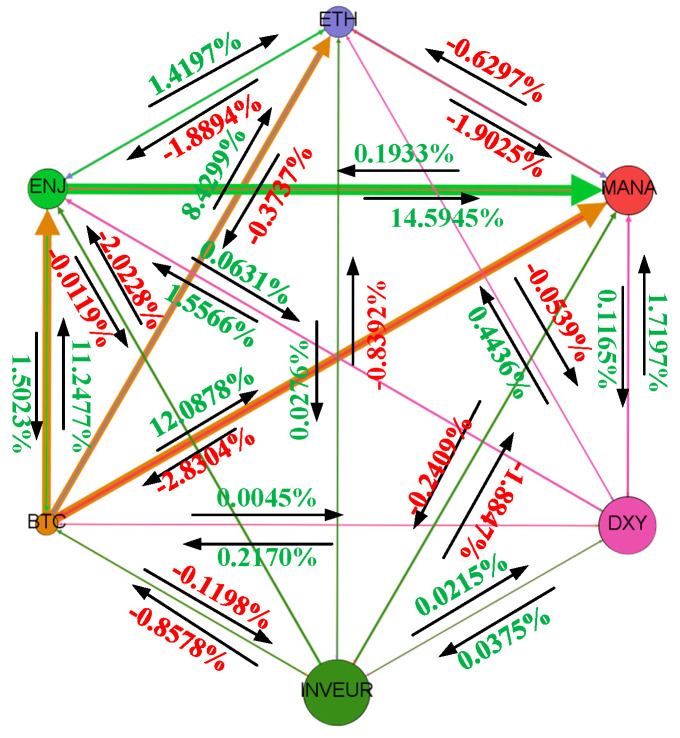
Average signed spillover network during the cryptocurrency slump period.

As shown in Figure 7, BTC exerts a relatively significant volatility spillover effect on the NFT market, in contrast to its role as a recipient of volatility spillovers in the full-sample analysis. This result is also consistent with the earlier statistical finding that the decline in cryptocurrency prices was accompanied by the depreciation of the NFT market. Since the sample period captures a period of cryptocurrency slump, the impact of cryptocurrency market fluctuations on the foreign exchange market remains extremely weak during this period, and the sign changes that exist are only accompanied by weak numerical switching.

### 5.5. Posterior Inference and Robustness Checks

The baseline analysis reveals substantial event-phase variation in signed spillovers across the cryptocurrency, NFT, and foreign exchange markets. To further assess the reliability of these findings, this subsection reports posterior inference and a series of robustness checks. Specifically, we examine the sensitivity of the main results to alternative crisis-window definitions, alternative lag structures, weekly calendar alignment, and alternative filtering choices.

#### 5.5.1. Alternative Crisis-Window Definitions

We test whether the COVID-19 evidence is sensitive to how the event window is defined. As reported in Table 3, the main pattern remains stable across narrow, baseline, and wide specifications: BTC continues to display a marked decline in cumulative net signed spillovers during the outbreak phase, whereas ENJ and MANA remain on the positive side. For example, the posterior mean difference for BTC ranges from about −30 to −36 across the three windows, while that for ENJ remains around 16 to 22. Although the MANA effect becomes somewhat weaker under the widest specification, its direction does not reverse. Overall, these results suggest that the main COVID-19 findings are not driven by an arbitrary choice of crisis-window length.

#### 5.5.2. Alternative Lag Structures

**Figure 8:**
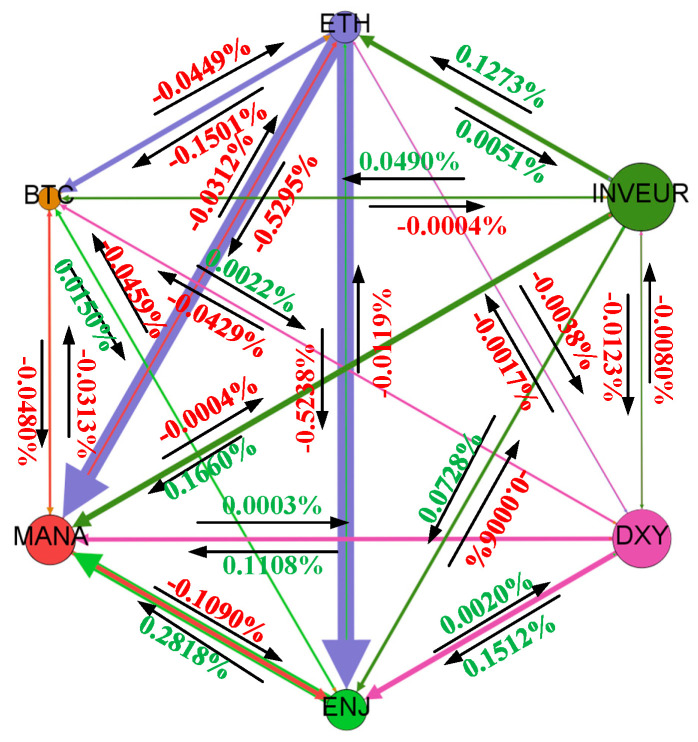
Average signed spillover network under the lag-2 specification.

Figure 8 shows that the lag-2 specification leaves the overall signed spillover structure largely unchanged. The main directional relations across the cryptocurrency, NFT, and foreign exchange markets are still visible, and no substantial structural reversal emerges when additional lag dynamics are introduced. This pattern indicates that the baseline network evidence is not overly sensitive to the lag specification.

#### 5.5.3. Weekly Calendar Alignment

Given the differences in trading frequency across cryptocurrency and foreign exchange markets, we further re-estimate the event-phase spillovers using a weekly common calendar. The results in Table 4 preserve the main pattern documented in the baseline specification: during the COVID-19 phase, BTC remains associated with a decline in cumulative net signed spillovers, whereas ENJ and MANA remain positive. Although temporal aggregation compresses the magnitude of the estimates, it does not alter their direction. This indicates that the baseline evidence captures a persistent cross-market transmission pattern rather than a feature tied to a particular daily alignment rule.

#### 5.5.4. Alternative Filtering Choices

To examine whether the main findings depend on the transformation of the original series, we replace the filtered baseline series with raw log returns computed directly from the price data. Table 5 shows that the central COVID-phase pattern remains intact under this alternative specification: BTC continues to exhibit a negative change in cumulative net signed spillovers, whereas ENJ and MANA remain positive. The numerical scale differs from that of the filtered specification, which is expected given the difference in data construction, but the direction of the main effects is preserved. This suggests that the core COVID-phase evidence reflects a stable signed transmission pattern across markets rather than a result driven by a particular filtering procedure.

## 6. Conclusions

In recent years, the increasing integration of finance and the Internet has drawn growing attention from markets and investors to the development of cryptocurrencies. As a result, the linkages among the cryptocurrency market, the NFT market, and the foreign exchange market have become an important topic of analysis. This paper uses historical decomposition to introduce sign as the fifth dimension of the connectedness network among the cryptocurrency market, the NFT market, and the foreign exchange market. Throughout the sample period, the signs of the spillover relationship between indicators for each market are time-varying. Compared with spillover magnitude, sign reversals occur more frequently across periods. In particular, the signed spillover from the foreign exchange market to the NFT market is relatively strong, whereas the spillover from the cryptocurrency and NFT markets to the foreign exchange market remains comparatively weak. This result indicates that sign variation provides additional information on cross-market transmission beyond the magnitude of spillovers alone.

To further analyze the signed network topology of each market, we use the average values over the sample period to examine the spillover effects of each market index. The average network indicates that the foreign exchange market exerts a negative signed spillover effect on the cryptocurrency market. By contrast, the cryptocurrency market exerts only weak spillovers on other markets and mainly acts as a recipient of volatility spillovers. In addition, the NFT market has a relatively significant negative spillover effect on the cryptocurrency market, consistent with its gradual maturity in the later period and reduced dependence on the cryptocurrency market. Finally, in periods of pronounced market turbulence, such as the outbreak period and the cryptocurrency slump period, the cryptocurrency market represented by BTC changed from a receiver of volatility spillover to a transmitter. From the perspective of the sign, spillover magnitudes vary little across periods, whereas the signs of spillover effects change more substantially.

## Figures and Tables

**Table 1:** The signed spillover matrix.

	$Y_{1}$	$Y_{2}$	⋯	$Y_{N}$	FROM
$Y_{1}$	$d_{11}^{T,z}$	$d_{12}^{T,z}$	⋯	$d_{1N}^{T,z}$	$\sum_{i=1}^{N}d_{1i}^{T,z},i\neq 1$
$Y_{2}$	$d_{21}^{T,z}$	$d_{22}^{T,z}$	⋯	$d_{N2}^{T,z}$	$\sum_{i=1}^{N}d_{2i}^{T,z},i\neq 2$
⋯	⋯	⋯	⋯	⋯	⋯
$Y_{N}$	$d_{N1}^{T,z}$	$d_{N2}^{T,z}$	⋯	$d_{NN}^{T,z}$	$\sum_{i=1}^{N}d_{Ni}^{T,z},i\neq N$
TO	$\sum_{j=1}^{N}d_{j1}^{T,z},j\neq 1$	$\sum_{j=1}^{N}d_{j2}^{T,z},j\neq 2$	⋯	$\sum_{j=1}^{N}d_{jN}^{T,z},j\neq N$	$S^{T,z}$

**Table 2:** Stationarity test results.

	Original Series		Cycle Component	
	ADF	PP	ADF	PP
Intercept (BTC) Intercept and trend None	0.4692 0.8444 0.5552	0.4567 0.8350 0.5476	0.0012 *** 0.0074 *** 0.0001 ***	0.0013 *** 0.0079 *** 0.0001 ***
Intercept (ETH) Intercept and trend None	0.4399 0.8187 0.4759	0.4401 0.8186 0.4780	0.0001 *** 0.0008 *** 0.0000 ***	0.0002 *** 0.0012 *** 0.0000 ***
Intercept (ENJ) Intercept and trend None	0.3093 0.6758 0.1748	0.3202 0.6898 0.1854	0.0054 *** 0.0279 ** 0.0003 ***	0.0058 *** 0.0299 ** 0.0003 ***
Intercept (MANA) Intercept and trend None	0.2740 0.6091 0.1388	0.2906 0.6333 0.1388	0.0014 *** 0.0084 *** 0.0001 ***	0.0020 *** 0.0117 ** 0.0001 ***
Intercept (DXY) Intercept and trend None	0.7333 0.7951 0.7860	0.7512 0.8123 0.7921	0.0005 *** 0.0037 *** 0.0000 ***	0.0005 *** 0.0033 *** 0.0000 ***
Intercept (INVEUR) Intercept and trend None	0.9574 0.8836 0.9978	0.9721 0.9261 0.9991	0.0000 *** 0.0000 *** 0.0000 ***	0.0000 *** 0.0000 *** 0.0000 ***

Note: ** and *** denote rejection of the null hypothesis at the 5% and 1% significance levels, respectively.

**Table 3:** Posterior inference under alternative crisis-window definitions.

Variable	Narrow Window (30 d)	Baseline Window (40 d)	Wide Window (45 d)
BTC	Δ = −29.48 [−40.78, −17.78] *p* (Δ < 0) = 1.000	Δ = −34.92 [−51.65, −17.48] *p* (Δ < 0) = 1.000	Δ = −35.74 [−54.25, −15.68] *p* (Δ < 0) = 1.000
ENJ	Δ = 15.88 [8.86, 24.91] *p* (Δ > 0) = 1.000	Δ = 20.73 [10.24, 34.39] *p* (Δ > 0) = 1.000	Δ = 22.39 [9.41, 38.66] *p* (Δ > 0) = 1.000
MANA	Δ = 14.78 [6.43, 21.05] *p* (Δ > 0) = 1.000	Δ = 14.90 [1.85, 23.99] *p* (Δ > 0) = 0.987	Δ = 14.59 [−0.01, 25.43] *p* (Δ > 0) = 0.973

Notes: Entries report posterior mean differences in cumulative net signed spillovers (event phase minus pre-event benchmark) for the COVID-19 outbreak window. Accordingly, 95% credible intervals are reported in brackets.

**Table 4:** Weekly calendar alignment robustness.

Variable	Daily Common-Calendar Baseline	Weekly Common-Calendar
BTC	Δ = −34.92 [−51.65, −17.48] *p* (Δ < 0) = 1.000	Δ = −5.94 [−8.15, −3.63] *p* (Δ < 0) = 1.000
ENJ	Δ = 20.73 [10.24, 34.39] *p* (Δ > 0) = 1.000	Δ = 2.74 [1.24, 4.31] *p* (Δ > 0) = 1.000
MANA	Δ = 14.90 [1.85, 23.99] *p* (Δ > 0) = 0.987	Δ = 3.65 [2.22, 5.12] *p* (Δ > 0) = 1.000

Notes: The weekly specification uses the last common observation of each week. Entries report posterior mean differences in cumulative net signed spillovers (event phase minus pre-event benchmark) for the COVID-19 outbreak window. Accordingly, 95% credible intervals are reported in brackets.

**Table 5:** Posterior inference under alternative filtering choices.

Variable	Filtered Baseline	Raw Log Returns
BTC	Δ = −34.92 [−51.65, −17.48] *p* (Δ < 0) = 1.000	Δ = −0.34 [−0.42, −0.24] *p* (Δ < 0) = 1.000
ENJ	Δ = 20.73 [10.24, 34.39] *p* (Δ > 0) = 1.000	Δ = 0.23 [0.17, 0.30] *p* (Δ > 0) = 1.000
MANA	Δ = 14.90 [1.85, 23.99] *p* (Δ > 0) = 0.987	Δ = 0.25 [0.21, 0.30] *p* (Δ > 0) = 1.000

Notes: The filtered baseline uses the cycle-based series in the main specification, whereas the alternative specification uses raw log returns computed directly from the original price series. Entries report posterior mean differences in cumulative net signed spillovers (event phase minus pre-event benchmark) for the COVID-19 outbreak window. Accordingly, 95% credible intervals are reported in brackets.

## Data Availability

The data used in this study are derived from publicly available market databases (cryptocurrency prices, NFT token prices, and foreign exchange indices). The processed datasets and analysis codes are available from the corresponding author upon reasonable request.
